# Emergency call utilization over a 10-years period: an observational study in Region Zealand, Denmark, 2013–2022

**DOI:** 10.1186/s13049-024-01307-w

**Published:** 2024-12-18

**Authors:** Thea Palsgaard Møller, Josefine Tangen Jensen, Annette Kjær Ersbøll, Stig Nikolaj Fasmer Blomberg, Helle Collatz Christensen

**Affiliations:** 1 Prehospital Center Region Zealand, Ringstedgade 61, 13., 4700 Næstved, Denmark; 2 Department of Anesthesiology and Intensive Care Medicine, Holbæk Hospital, Holbæk, Denmark; 3 Department of Clinical Medicine, University of Copenhagen, Copenhagen, Denmark; 4 National Institute of Public Health, University of Southern Denmark, Odense, Denmark; 5 Emergency Medical Services, Capital Region of Denmark, Copenhagen University Hospital, Ballerup, Denmark

**Keywords:** Emergency calls, Emergency medical dispatching, Emergency medical services, System improvement

## Abstract

**Background:**

Improving prehospital emergency care requires a comprehensive understanding of the efficiency of emergency medical services and demand fluctuations. The medical emergency call is the primary contact between citizens and the emergency medical dispatch center, serving as the gateway to accessing emergency assistance. This study aimed to characterize the emergency call population and analyze the development of emergency call utilization in Region Zealand in Denmark during a 10-years period.

**Methods:**

This was an observational register-based study of administrative data from the emergency medical dispatch center in Region Zealand. Data was collected from 1 January 2013 to 31 December 2022. All unique emergency calls from residents to the emergency number “1-1-2” were included. Descriptive analyses were used to characterize the study population. Poisson regression models were used to calculate ratio estimates for the association between years and hospital catchment areas, using the incidence rate of emergency calls as outcome measure.

**Results:**

A total of 641,457 emergency calls were included. A significant increase in the total number of emergency calls was found, with an increase from 58,454 annual calls to 80,819 calls over the study period. The incidence rate per 1000 residents per year increased from 71.1 to 95.2, a 35% increase. The southern part of the region had significantly more emergency calls per 1000 residents per year during the study period compared to the eastern part of the region (IRR 1.70). Demographically, males comprised 52.3% of cases, and patients aged 65 and older represented 48.2% of calls. Emergency calls were “Emergency level A” in 45.5% and “Emergency level B” in 39.1%. In 22.3% of cases, the emergency call was categorized as “Unclear problem.” The most frequent categories were “chest pain” (12.7%), “impaired consciousness” (9.6%), “breathing difficulties” (8.8%), “accidents” (7.9%), and “minor injuries” (7.6%).

**Conclusions:**

The study revealed a significant increase in emergency calls, both in absolute numbers and per 1000 residents per year, indicating growing demand for emergency care, along with a surge in activity at the region's dispatch center. Regional disparities underscores the potential necessity for tailored developmental approaches over time.

## Background

Emergency medical services (EMS) play a critical role in providing timely and life-saving interventions to individuals in need of urgent medical assistance. The effectiveness of the chain of pre-hospital care is therefore of great interest. The initial point in this chain is the emergency call to an emergency medical dispatch center (EMDC). Here, the medical dispatcher must promptly identify life-threatening conditions [1, 2] and provide pre-arrival instructions for bystanders while simultaneously dispatching the appropriate emergency medical services to the patient. The efficiency and effectiveness of medical dispatching are crucial factors that directly impact on patient outcomes. Rapid recognition of out-of-hospital cardiac arrest (OHCA) during emergency calls has been associated with improved survival [3, 4], and recognition of stroke and acute coronary syndrome is associated with improved treatment course [5, 6].

Importantly, dispatchers play a crucial role that extends beyond responding to life-threatening emergencies. They also serve as gatekeepers to the healthcare system, managing ambulance resources efficiently by assessing the nature and urgency of each call. Ultimately, the emergency call serves as gatekeeper for admissions to emergency departments, where overcrowding is an increasing concern linked to adverse patient outcomes. The triage of emergency calls primarily contributes to input factors of overcrowding, such as EMS non conveyance [7], rising numbers of patients with urgent, complex, low-acuity, or elderly care needs [8–10]. However, overcrowding is also influenced by throughput factors, including staffing shortages, limited bed availability, and diagnostic delays, as well as output factors, such as challenges in transferring patients to inpatient beds [8–10].

Understanding the temporal development in utilization patterns for medical emergency calls is vital to gain insights into the needs of the residents and for optimizing prehospital emergency care. This study aimed to characterize the emergency call population and evaluate the development in the number of medical emergency calls in Region Zealand, Denmark over a 10-year period from 2013 to 2022.

## Methods

The study was an observational retrospective register-based study in a 10-year study period from 1 Januar 2013 to 31 December 2022.

Analyses were based on administrative data from the EMDC in Region Zealand. All calls from patients present in Region Zealand at the time of call were included.

### Setting

Denmark is a small country in Northern Europe and is home to nearly 6 million people. The country has since 2007 been divided into five administrative regions, each responsible for their own healthcare. Region Zealand has a population of approximately 850,000 citizens, with Roskilde, the largest city, housing just over 50,000 residents. In the rural areas the population density differs from 8 to 28 residents per km^2^ [11].

In Denmark, residents have free and open access to health care via a single emergency phone number (1–1–2) leading to a primary call center for emergency police, fire, or medical requests, manned by police or fire personnel. Medical emergency calls are then directed to one of five regional EMDCs, staffed by medical dispatchers who are either experienced nurses or paramedics with supplemental training in medical dispatching. The medical dispatchers use a national criteria-based dispatch tool called Danish Index for Emergency Care to prioritize and categorize the incoming calls into 38 main categories according to the presented clinical signs, symptoms or incident type and based on information about the character and severity of the problem, the dispatch system suggests a relevant ambulance response and pre-arrival instructions to the caller. The response level suggested by the dispatch tool can be “A” (immediate ambulance response with lights and siren); “B” (immediate ambulance response without lights and siren); “C” (non-urgent ambulance response with available appropriate resources); “D” (transport only, no need for treatment); or “E” (medical advice, referral to a general practitioner, etc.). Logistics dispatchers distribute the emergency response selected by the medical dispatcher and must simultaneously prioritize the vehicles in the two-tiered ambulance response system where there are physician (anesthesiologists)- and paramedic-staffed mobile critical care units in addition to ambulances staffed with paramedics and emergency medical technicians [12]. Despite use of the same dispatch system, dispatch criteria of the MCUs varies between regions. The EMS are supported by a national Helicopter Emergency Medical Service [13]. For non-urgent conditions requiring medical advice, a doctor-on-call service is available outside the regular operating hours of general practitioners.

### Study population

The study comprised patients of all ages for whom at least one emergency call had been performed in the study period. Patients were included several times if they had more than one call. Furthermore, patients were included regardless of their region of residence, to reflect the local EMS system load. Internal calls such as calls from ambulance personnel or personnel from nursing homes or general practice to the EMDC were excluded. If an incident had more than one call to the dispatch center, only the first call was kept for analysis. If there was more than one patient identified at an incident only the first patient was included in the analysis.

### Data management and data sources

The patients were identified through the administrative database in the EMDC in Region Zealand. Due to a shift in the organization's logistics management system in October 2017, two databases were integrated with consecutive continuous data that had slightly different data structures. The data in the older of the two systems were based on each dispatch separately and then linked with data from the call system. The later data were incident-based, and each call was linked to the subsequent EMS dispatches. The database contains the personal identification number, dispatch code (which includes the main category and the emergency priority level A-E), the provided ambulance response type and time stamps from the entire prehospital patient trajectory. Finally, the zip code of the incident was included in the dataset to construct a variable of the emergency hospital catchment area in the region. Data were stored in a legal and secure research data web application, from where data management and statistical analyses were performed. All data management and statistical analyses were performed in R v.4.4.0.

### Statistical analysis

**Fig. 1:**
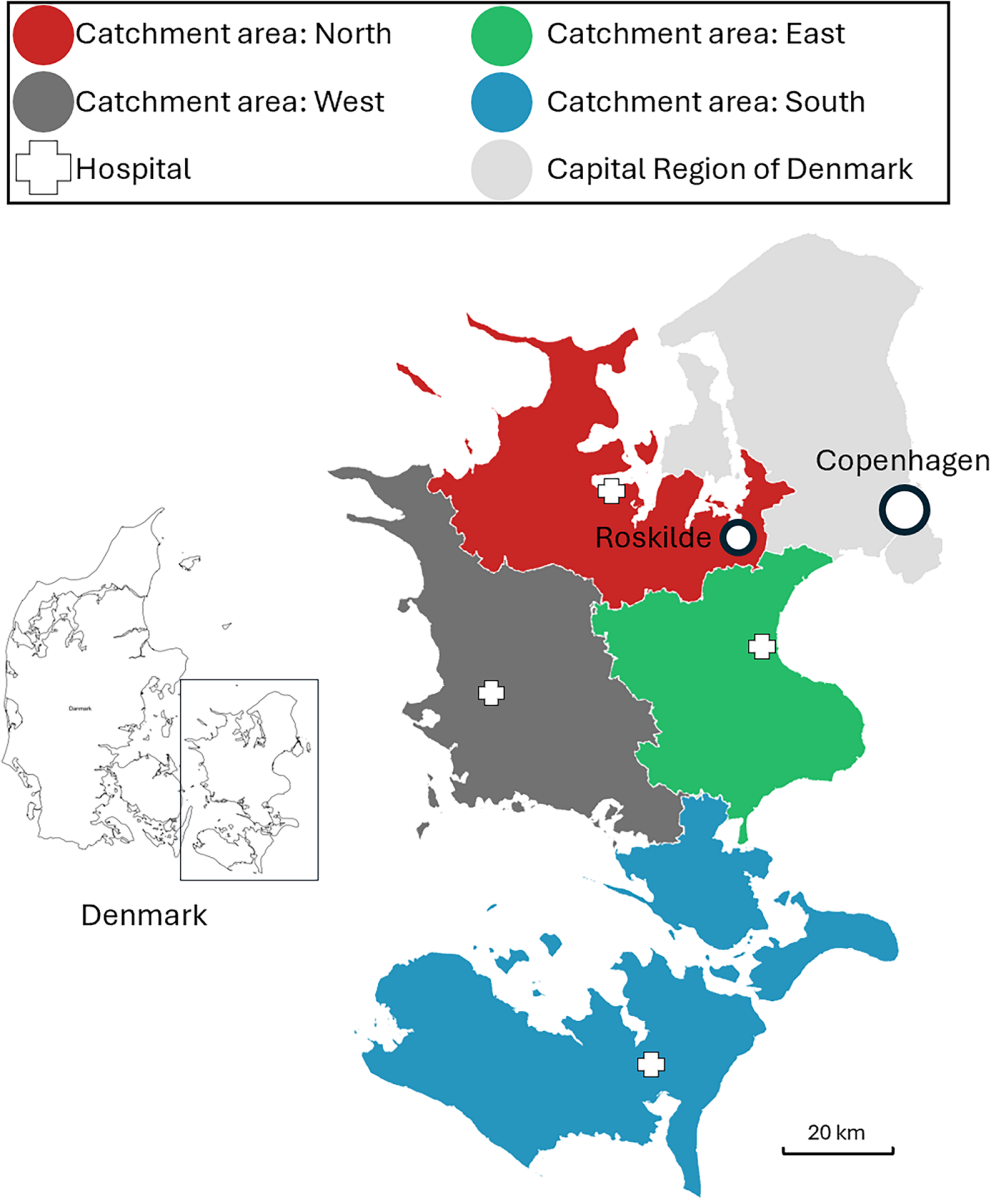
Map of Region Zealand illustrating the four catchment areas corresponding to the four hospitals with emergency departments in the region [20]

Descriptive analyses were performed of baseline characteristics of study participants by use of absolute numbers and percentages. A Poisson regression model of rates [14] was used to calculate ratio estimates for the association between years, hospital catchment areas and incidence rates (IR) of emergency calls per 1,000 residents per year. The median age was included in the analysis to adjust for the effect of increasing population age over time. The analyses were performed overall and stratified by hospital catchment area of each of the region’s four hospitals with emergency departments (Fig. 1). A *p*-value < 0.05 was considered statistically significant.

## Results

**Fig. 2:**
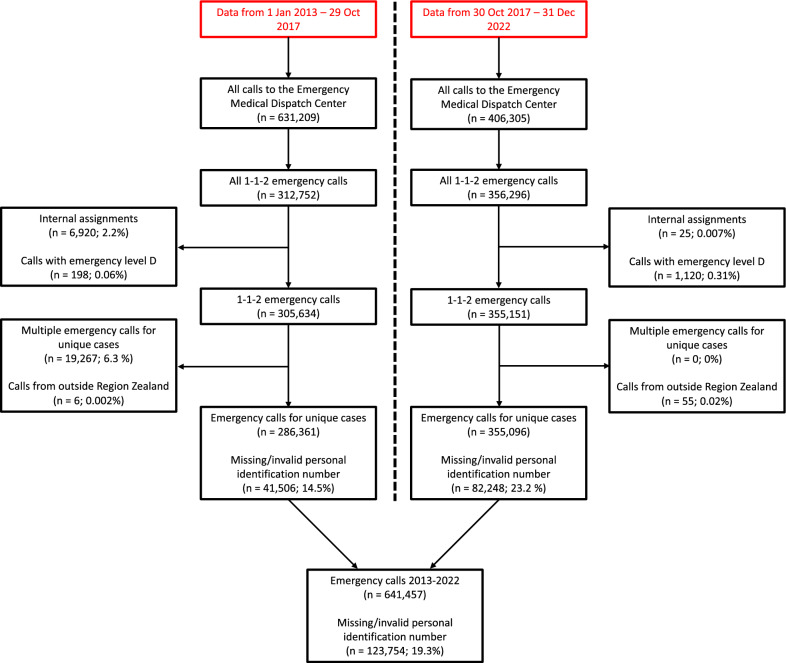
Data flowchart

A total of 641,457 emergency calls were included from the administrative data in the study period. These were included in the analysis of development in emergency calls. For a total of 123,754 cases, the personal identification number was not available (including information on sex and age), leaving 517,703 emergency calls for the population characterization (Fig. 2).

Demographically (Table 1), men accounted for 52.3% of the calls overall with a similar distribution across the four hospital catchment areas. Children aged 0–14 years accounted for 5.1% of cases, adults aged 25–64 years comprised 38.1% of cases, and adults aged 65 or more constituted 48.2% of cases. The southern part of the region had a higher proportion of elderly patients with 50.1% of the cases involving individuals aged 65 years and above.

**Table 1:** Characteristics of study population at inclusion overall and stratified by hospital catchment area

	Overall	Hospital catchment area			
		East	North	South	West
*Sex*					
Female, n (%)	246,878 (47.7)	60,819 (48.2)	61,771 (47.9)	57,568 (46.6)	66,720 (48.0)
Male, n (%)	270,825 (52.3)	65,398 (51.8)	67,250 (52.1)	65,838 (53.4)	72,339 (52.0)
*Age group*					
0–14 years, n (%)	26,481 (5.1)	7835 (6.2)	6555 (5.1)	4946 (4.0)	7145 (5.1)
15–24 years, n (%)	44,353 (8.6)	10,367 (8.2)	11,801 (9.1)	9220 (7.5)	12,965 (9.3)
25–64 years, n (%)	197,461 (38.1)	46,959 (37.2)	48,065 (37.3)	47,415 (38.4)	55,022 (39.6)
≥ 65 years, n (%)	249,408 (48.2)	61,056 (48.4)	62,600 (48.5)	61,825 (50.1)	63,927 (46.0)
*(missing, n)*	123,754	26,127	31,385	33,559	32,683

**Fig. 3:**
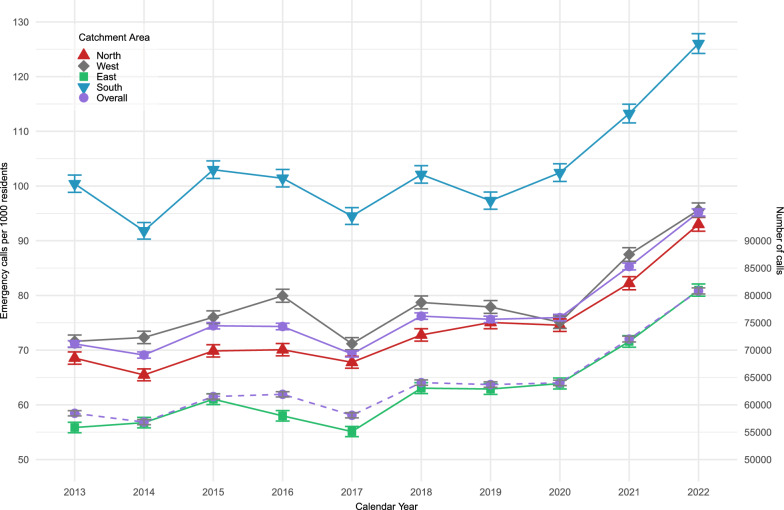
Emergency callrate per 1000 residents by hospital catchment area and total number of emergency calls (dotted line) in Region Zealand, 2013–2022

The total number of emergency calls increased significantly (Table 2 and Fig. 3) from 58,454 annual calls to 80,819 annual calls over the study period. The emergency calls per 1000 residents per year increased from 71.1 to 95.2 corresponding to a 35% increase (Fig. 3). The southern part of the region had significantly more emergency calls per 1000 residents per year during the study period compared to the eastern part of the region (IRR 1.70 (95% CI 1.68; 1.72).

**Table 2:** Emergency calls, callrate and classification according to Danish Index for Emergency Care. Overall and stratified by hospital catchment area

	Overall		Hospital catchment area				
			East	North	South		West
*Emergency calls, total*							
2013, n	58,454		13,059	14,450	15,545		15,400
2022, n (% compared to 2013)	80,819 (138%)		20,169 (154%)	20,686 (143%)	18,822 (121%)		21,142 (137%)
*Emergency calls/1000 residents/year*							
2013	71.1		55.9	68.5	100.4		71.6
Incidence Rate [95% CI]	[70.6; 71.7]		[54.9; 58.8]	[67.4; 69.7]	[98.8; 102.0]		[70.5; 72.8]
2022	95.2		81.0	93.0	126.0		95.6
Incidence Rate [95% CI]	[94.5; 95.8]		[79.9; 82.1]	[91.7; 94.3]	[124.2; 127.8]		[94.3; 96.9]
*Incidence rate ratio (IRR)*							
Reference year 2013	IRR 1.53		Reference	IRR 1.18	IRR 1.70		IRR 1.17
Incidence Rate Ratio [95% CI]	[1.47;1.58]			[1.17;1.19]	[1.68;1.72]		[1.15;1.19]
Emergency level provided*							
A	248,733 (45.5)		60,877 (45.8)	62,073 (45.1)	59,413 (45.7)		66,370 (45.4)
B	213,570 (39.1)		51,807 (39.0)	53,491 (38.9)	50,538 (38.9)		57,734 (39.5)
C	10,197 (1.9)		2454 (1.8)	2440 (1.8)	2549 (2.0)		2754 (1.9)
E	74,264 (13.6)		17,870 (13.4)	19,664 (14.3)	17,375 (13.4)		19,355 (13.2)
*(missing, n* = *94,693)*			*19,336*	*22,738*	*27,090*		*25,529*
Index Category*							
Unclear problem	122,071 (22.3)		29,027 (21.8)	31,001 (22.5)	29,232 (22.5)		32,811 (22.5)
Chest pain	69,272 (12.7)		16,964 (12.8)	16,958 (12.3)	17,034 (13.1)		18,316 (12.5)
Impaired consciousness, paralysis, dizziness	52,624 (9.6)		13,076 (9.8)	13,010 (9.5)	12,574 (9.7)		13,964 (9.6)
Breathing difficulties	48,084 (8.8)		11,276 (8.5)	11,652 (8.5)	12,710 (9.8)		12,446 (8.5)
Accidents	43,254 (7.9)		11,095 (8.3)	11,316 (8.2)	9605 (7.4)		11,238 (7.7)
Minor injuries	41,389 (7.6)		10,182 (7.7)	10,497 (7.6)	9581 (7.4)		11,129 (7.6)
Abdominal pain, back pain	34,199 (6.3)		8420 (6.3)	8034 (5.8)	8496 (6.5)		9249 (6.3)
*(missing, n* = *95,056)*			*19,420*	*22,826*	*27,174*		*25,636*

*According to Danish Index for Emergency Care [12]. IRR = Incidence rate ratio

**Fig. 4:**
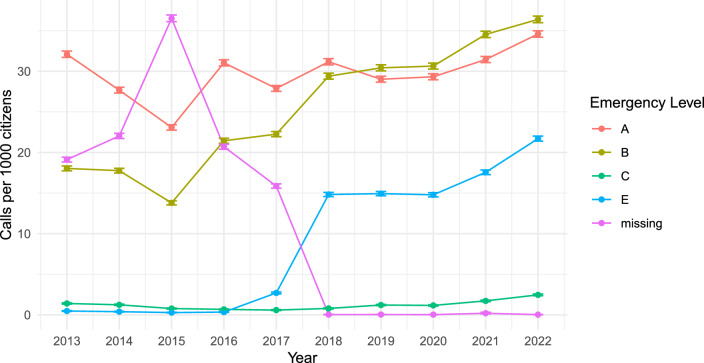
Emergency level registered per 1000 residents in Region Zealand, 2013–2022

The medical dispatchers assessed the urgency of calls as "Emergency level A" in 45.5% and "Emergency level B" in 39.1% of calls. Figure 4 shows the emergency levels registered per 1000 residents in Region Zealand in 2013–2022.

**Fig. 5:**
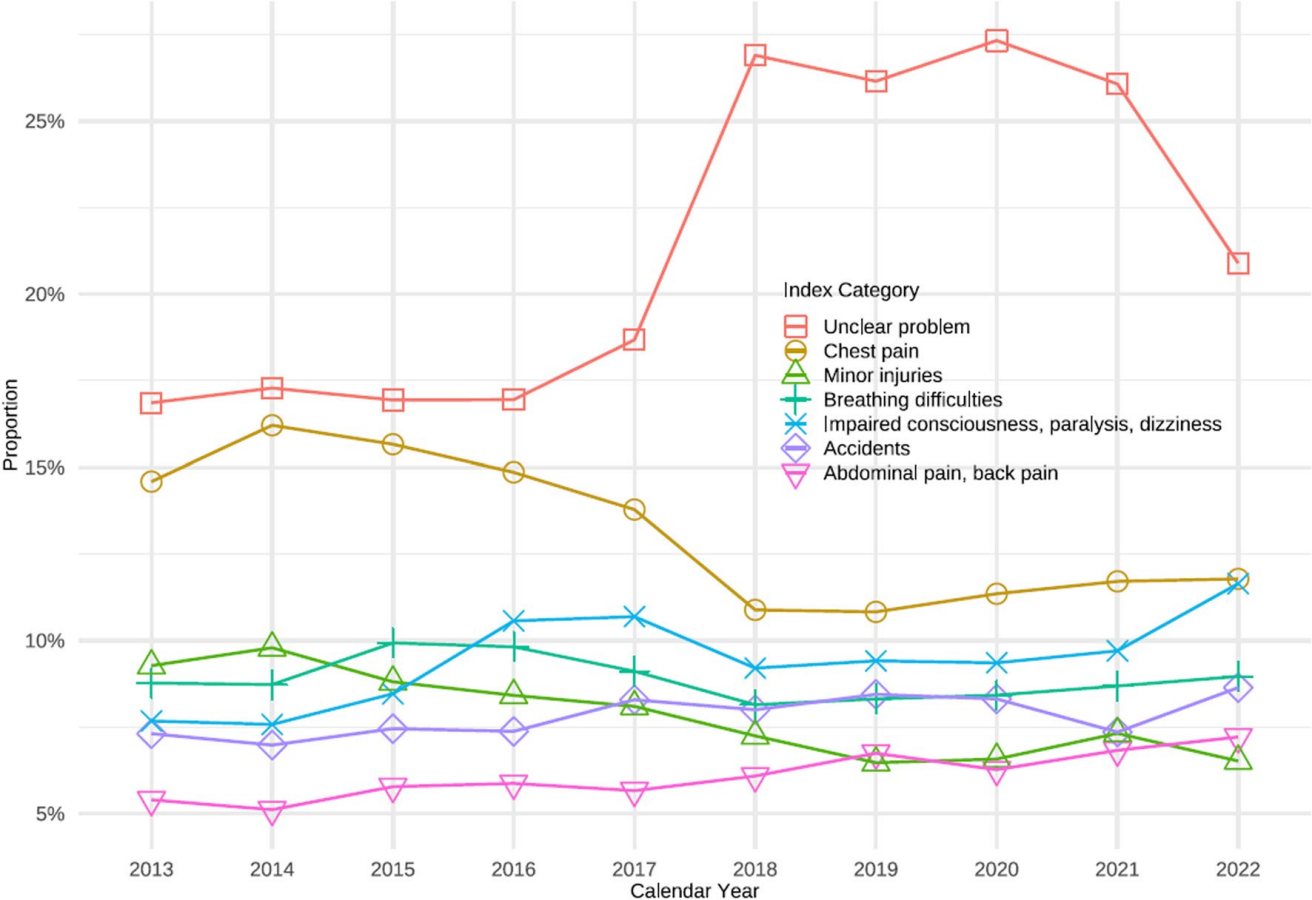
Proportion of the most common Danish Index categories by calendar year 2013–2022

In terms of emergency call categorization, the most frequently assigned category was “Unclear problem” accounting for 22.3% of cases. The most frequently reported specific causes were "chest pain" (12.7%), "impaired consciousness" (9.6%), "breathing difficulties" (8.8%), "accidents" (7.9%), and "minor injuries" (8.6%). Table 2 and Fig. 5 shows emergency call utilization overall and within hospital catchment areas.

## Discussion

The main findings of this study were a significant increase in both the total number and the IR of emergency calls over the study period. A geographical variation in the IR was found. Men made up just over half of the cases, and the elderly constituted the largest portion of the study population, with some variation across different areas. The most common reasons for emergency calls were unclear problems, chest pain, breathing difficulties, neurological issues, accidents, and minor injuries.

### Variations in incidence rate

The notable increase in the volume of emergency calls to the EMDC over the study period underscores several important considerations. The rising trend in emergency calls reflects an escalating demand for EMS within the region. This trend has also been found in other studies. Herr et al. [15] found an overall increase in emergency calls of 9.1% over a three-year period from 2018 to 2021 in Berlin. Campagna et al. [16] found a 34% increase in total number of calls over a four-year period from 2013 to 2017 in Italy, and at the same time emergency call IR increased from 55 to 102 calls/1000 residents/year. This increased demand could stem from various factors, including access to other medical services or changes in healthcare utilization patterns. Thus, a new report found a significant decrease in available general practitioners in Region Zealand during the last 15 years [17]. Moreover, a recent review found that systemic barriers to timely primary care, coupled with patient perceptions of urgency and reliance on emergency services for immediate assistance, lead many individuals to default to ambulance use even for non-critical conditions [18].

### Geographical variations

The finding of geographic variations in numbers of emergency calls also merit attention, as they may reflect differences in population density, socioeconomic factors, and healthcare infrastructure across different regions within the study area. The findings are comparable with a study by Hegenberg et al. [19] who found a difference in emergency call IR between rural municipalities and larger cities, with respective 42.8 and 80.7 emergencies/1,000 residents/year suggesting that future EMS planning should be targeted to regional characteristics. In addition, a newly published study from our group, which examines the increase in ambulance missions alongside trends in age composition and comorbidity across the four geographic areas analyzed in this article, found a larger increase in the proportion of elderly in the southern part of the region, which may contribute to the findings in this study [20].

Furthermore, numbers from Statistics Denmark show that the municipalities corresponding to the southern catchment area have a lower level of employment, a lower average income, fewer highly educated people, and a shorter lifespan, pointing to overall poorer health [11]. Finally, a recent report on the region's health profile has shown that there are more citizens with long-term illnesses in the southern part of the area [21].

### Contact causes

The pattern of contact causes to the EMS through emergency calls are similar to findings in other studies. In a previous investigation in the Capital Region of Denmark, “unclear problem” was the most frequent cause of contact, accounting for 19% of cases and the five most common known causes were categorized as “wounds, fractures, minor injuries” (13%), “chest pain/heart disease” (11%), “accidents” (9%), “intoxication, poisoning, drug overdose” (8%), and “breathing difficulties” (7%) [12]. In addition, 81% of calls were assigned Emergency levels A and B, which is comparable to our findings.

The categorization of causes of emergency calls has been shown to be associated with mortality. One study found that “unclear problem” was associated with higher mortality for emergency level B calls compared to those with a known cause [22]. Another study found that “unclear problem” was among the three most deadly dispatch categories of causes during emergency calls, along with “breathing difficulties” and “unconscious adult/possible cardiac arrest" [23]. Both studies highlight the need for improvement of emergency call handling and the underlying supportive decision tools. Educational interventions at the dispatch centers may improve the procedure, as shown in a recent study, where categorization of the cause as “unclear problem” by medical dispatchers was markedly reduced after a period of increased focus on specific categorization of causes of emergency calls [24].

Overall, these studies emphasize the need for more accurate dispatch systems to ensure efficient use of EMS resources and optimal patient outcomes. Accurate dispatch is crucial for the effective utilization of limited EMS resources. Various studies have evaluated the performance of different dispatch systems. A systematic review aimed to assess the evidence for medical dispatch systems' accuracy in identifying the level of acuity and specific conditions. The review included 18 studies, finding a very low to low level of evidence for dispatch system accuracy [25]. One observational study of trauma patients transported to major trauma centers assessed dispatch accuracy by evaluating the sensitivity, under- and overtriage rates. While most patients requiring specialized care were assigned the highest priority by dispatch centers and EMS professionals, significant undertriage rates existed at the same time, indicating suboptimal prehospital triage accuracy for trauma patients [26]. Another study examined the Geneva emergency medical dispatch system (EMD), revealing good specificity but low sensitivity in dispatching emergency physicians. This study suggested using the dichotomy between immediate life-threatening emergencies and other emergencies as a reference standard for future performance assessments [27]. Research in Norway validated the Norwegian dispatch tool as an effective predictor of patients not needing pre-hospital interventions, particularly for low-acuity cases. However, it also highlighted the challenge of evaluating adherence to protocols due to the necessity of medical expertise in the triage process [28]. Lastly, a study analyzing 6,416 EMS dispatches found discrepancies between EMD and EMS priorities, with the EMD often overestimating the urgency of dispatches. While the EMD was consistent in identifying non-urgent cases, its overestimation of urgent cases could impact the availability of EMS for truly urgent missions, suggesting a need for improved accuracy to enhance EMS efficiency [29]. Notably, the dispatchers in this study were highly trained nurses or paramedics, reflecting a high level of medical expertise. This high level of competence is further supported by their continuous professional development, ensuring their skills and knowledge remain aligned with evolving standards in emergency medical care, which should be considered.

## Implications

Overall, the findings underscore the dynamic nature of emergency call volumes and the multifaceted challenges associated with managing and responding to increasing demand for EMS. By elucidating the trends and patterns in emergency call utilization, this study provides valuable insights for informing policy decisions, resource allocation strategies, and future research efforts aimed at optimizing emergency care delivery and ultimately improving patient outcomes.

Knowledge of the use of the emergency system is essential for both residents and decision-makers in a time of limited resources. Knowledge of temporal development can help to foresee necessary organizational changes in the future. The finding of the increase in emergency call utilization indicate that the staffing of the emergency center may need to be expanded in the future. Finally, the results highlight a possible need for in-depth studies on the effectiveness of the system, especially for modifiable factors such as the dispatch system provided. Moreover, the observed increase in numbers of emergency calls raises concerns about the capacity of the EMS system to manage the escalating workload. An increase in call volume can strain resources, potentially leading to longer response times, increased waiting times for ambulances, and heightened risk for adverse patient outcomes.

An important implication to consider is the potential role of telephone care assessment systems and medical helpline services in reducing the volume of emergency calls and the workload for dispatchers and EMS. Integrating these services more into the healthcare system could potentially streamline the use of emergency resources. Additionally, raising public awareness about when to use specific services, such as distinguishing between non-urgent helplines and emergency numbers, is crucial for ensuring appropriate service utilization and reducing unnecessary strain on emergency response systems.

Addressing these challenges requires proactive measures such as enhancing resource allocation strategies, optimizing dispatch protocols, and adjusting EMS capacity to ensure timely and efficient emergency response.

## Limitations

To evaluate the development over a ten-year period, we had to use two datasets with a slightly different structure. This may reflect some of the results, especially major changes around the year 2017. The causes and emergency levels were extracted from a specific variable from the EMS database. The change in 2017 coincided with an update in EMS data registration, which may be interrelated. In Fig. 4, the data show a significant reduction in missing entries around 2017, suggesting improved registration practices. This improvement could partly explain the observed increase in cases categorized as “unclear problem.” It is likely that some cases previously classified as missing were reclassified as “unclear problem” following the changes in registration.

Data in this study were all registered electronically, but the specific variable concerning cause and emergency priority level, reflects medical dispatchers’ decisions during an emergency call according to the flowchart in their supportive priority tool. This may introduce classification bias. Further there is a large proportion of missingness in the personal identification number variable, which affects the analysis of age and gender, as cases with missingness are mostly completed without patient contact, and therefore not representative for the population. Finally, the study was conducted in Region Zealand, and the results may reflect a pattern for a mixed urban and rural area. This might decrease the generalizability of the identified contact patterns from a national and international perspective.

## Conclusions

The study identified a significant increase in emergency calls, both in absolute numbers and per 1000 residents per year. The results suggest an increased demand for emergency care among residents, along with a corresponding increase in workload at the region's dispatch center. Additionally, the identification of regional disparities underscores the potential necessity for tailored developmental approaches over time.

## Data Availability

No datasets were generated or analysed during the current study.

## Abbreviations

CI: Confidence interval
EMD: Emergency medical dispatch
EMDC: Emergency medical dispatch center
EMS: Emergency medical services
IR: Incidence rate
IRR: Incidence rate ratio
